# Risk of lymphoma subtypes by occupational exposure in Southern Italy

**DOI:** 10.1186/s12995-017-0177-2

**Published:** 2017-11-23

**Authors:** Giovanni Maria Ferri, Giorgina Specchia, Patrizio Mazza, Giuseppe Ingravallo, Graziana Intranuovo, Chiara Monica Guastadisegno, Maria Luisa Congedo, Gianfranco Lagioia, Maria Cristina Loparco, Annamaria Giordano, Tommasina Perrone, Francesco Guadio, Caterina Spinosa, Carla Minoia, Lucia D’Onghia, Michela Strusi, Vincenzo Corrado, Domenica Cavone, Luigi Vimercati, Nunzia Schiavulli, Pierluigi Cocco

**Affiliations:** 1 Department of Interdisciplinary Medicine (DIM), Section “B. Ramazzini”, Regional University Hospital “Policlinico - Giovanni XXIII°”, Unit of Occupational Medicine, University of Bari, Piazza G. Cesare, 11, 70124 Bari, Italy; 2 Department of Emergency and Transplantation (DETO), Regional Universitary Hospital “Policlinico - Giovanni XXIII°, Unit of Hematology, University of Bari, Piazza G. Cesare, 11, 70124 Bari, Italy; 3 ASL Taranto, Moscati Hospital, Unity of Haematology, Via Paisiello 1, 74100 Taranto, Italy; 4 Department of Emergency and Transplantation (DETO), Regional University Hospital “Policlinico - Giovanni XXIII° ”, Unit of Pathology, University of Bari, Piazza G. Cesare, 11, 70124 Bari, Italy; 5 Department of Public Health, Clinical & Molecular Medicine, Occupational Health Section, University of Cagliari, 09100 Cagliari, Italy; 6 Interdisciplinary Department of Medicine (DIM), University Hospital. Policlinico-Giovanni XXIII, University of Bari, Piazza Giulio Cesare, 11, 70124 Bari, Italy

**Keywords:** Lymphomas, Occupational exposure, CAREX matrix, Pesticides, B-cell lymphoma subtypes, Case–control study

## Abstract

**Background:**

Occupational exposure is known to play a role in the aetiology of lymphomas. The aim of the present work was to explore the occupational risk of the major B-cell lymphoma subtypes using a case–control study design.

**Methods:**

From 2009 to 2014, we recruited 158 lymphoma cases and 76 controls in the provinces of Bari and Taranto (Apulia, Southern Italy). A retrospective assessment of occupational exposure based on complete work histories and the Carcinogen Exposure (CAREX) job-exposure matrix was performed.

**Results:**

After adjusting for major confounding factors, farmers showed an increased risk of diffuse large B-cell lymphoma (DLBCL) [odds ratio (OR) = 10.9 (2.3–51.6)] and multiple myeloma (MM) [OR = 16.5 (1.4–195.7)]; exposure to the fungicide Captafol was significantly associated with risk of non-Hodgkin lymphoma (NHL) [OR = 2.6 (1.1–8.2)], particularly with the risk of DLBCL [OR = 5.3 (1.6–17.3)].

**Conclusions:**

Agricultural activity seems to be a risk factor for developing lymphoma subtypes, particularly DLBCL, in the provinces of Bari and Taranto (Apulia Region, Southern Italy). Exposure to the pesticides Captafol, Paraquat and Radon might be implicated.

**Trial registration:**

Protocol number UNIBA 2207WEJLZB_004 registered 22/09/2008.

## Background

According to 2007–2010 data from the Italian Association of Cancer Registries/Associazione Italiana Registri Tumori (AIRTUM), the estimated standardized incidence rate of haemolymphopoietic cancers was lower in Southern than in Northern Italy; this difference was more significant for non-Hodgkin lymphoma (NHL), multiple myeloma (MM) and chronic lymphocytic leukaemia (CLL) and less significant for Hodgkin lymphomas (HL) [1]. The Apulia areas of reference were the provinces of Bari and Taranto. The estimated standard rates of incidence (× 10,000) of CLL in Taranto Province were 12.8 for males and 12.1 for females. In Bari Province, the rates were 18.1 for males and 11.9 for females. In the Apulia region, they were 14.7 for males and 9.1 for females. The estimated standard rates of incidence of HL were 3.9 for males and 4.1 for females in Taranto Province, 4.5 for males and 5.8 for females in Bari Province and 3.8 for males and 3.7 for females in the Apulia region. The estimated standard rates of incidence of NHL were 14.6 for males and 9.3 for females in Taranto Province, 20.7 for males and 15.0 for females in Bari Province, and 14.7 for males and 10.6 for females in the Apulia region. The estimated standard rates of incidence of MM were 5.2 for males and 4.1 for females in Taranto Province, 7.4 for males and 6.1 for females in Bari Province and 6.3 for males and 4.8 for females in the Apulia region [2].

The north/south gradients might suggest various causes, including a lesser prevalence of exposure to occupational and environmental carcinogens and to tobacco smoking and a higher prevalence of protective factors such as healthier food habits and a younger age at first pregnancy in Southern Italy. Typical Mediterranean diet items, in particular fruits and vegetables, showed an inverse association with NHL risk [3]. Furthermore, a case–control study of the risk of MM among women in the International Multiple Myeloma Consortium [4] showed a decreased risk of follicular lymphoma (FL) with an increasing number of pregnancies and an association between FL and hormonal contraception [5]. A lower rate of participation in cancer screening programmes in the southern regions of Italy might also reflect a lower detection rate. In terms of mortality, the previously reported North–south gradient has been gradually decreasing in recent years [1]. The observed difference in Taranto and Bari could be explained by different regional industrial activities and other studied factors.

The role of chemical agents (pesticides, solvents), ultraviolet radiation and infectious agents has been extensively studied [6–9].

Male farmers tend to have lower overall cancer incidence and mortality, which might be due to their lower smoking prevalence and increased physical activity [10–13]. However, the risk of certain cancers, including lymphohaematopoietic cancer, has been reported to be increased among farmers [10, 14, 15].

Rieutort et al. found that NHL was associated with various occupational activities and exposures; among them, those involving agricultural or industrial sectors and solvents or pesticides were highlighted, with the highest number of publications and the strongest association with NHL risk [16].

The aim of this case–control study is to assess the association between occupational activities and lymphoma subtypes and to study the occupational risk factors involved in the occurrence of those lymphoma subtypes in the provinces of Taranto and Bari in the Apulia Region (Southern Italy) using the “Carcinogen Exposure” (CAREX) matrix for assessing occupational carcinogen exposure.

## Methods

This study reports the activity of a unit of “The Multicentre Italian Study on Gene-Environment Interactions in Lymphoma Etiology” financed by the Italian Projects of National Interest (PRIN) and the Italian Association for Cancer Research (AIRC) and participated in the InterLymph Consortium initiated by the U.S. National Cancer Institute from 2009 to 2014 and in the Genome-Wide Association Study (GWAS) project. The recruitment of patients was performed in the haematology division of the University Hospital of Bari and in the haematology division of the “Moscati” Hospital of Taranto in the Apulia region (Southern Italy). The study was coordinated by the Occupational Section of the Interdisciplinary Department of Medicine (DIM), University of Bari, Italy.

### Study sample

One hundred fifty-eight incident cases of lymphoma first diagnosed during the study period were included: 30 cases of HL and 128 cases of NHL [35 cases of diffuse large B-cell lymphoma (DLBCL), 26 FL, 42 CLL, 3 mantle cell lymphoma, 8 small B-cell lymphoma (SBCL), 11 MM, and 3 mucosa-associated lymphoma tissue (MALT)]. For each case, the diagnosis was reviewed and classified using the 2008 World Health Organization (WHO) classification of lymphoma [17]. During the same period, 76 controls were enrolled and were selected by a matching method (age at first diagnosis, sex and residence); however, due to the small number of controls, no matching analysis was performed. The selection of population controls was performed by accessing the assisted regional register and identifying a list of subjects of the same sex, same age class, and same province of residence (provinces of Bari and Taranto) as the cases. Subsequently, using a simple randomization method, controls were identified and contacted by phone. Outpatient controls were recruited at the ophthalmological and orthopaedic outpatient clinic of Bari Hospital and Taranto Radiology, after the inclusion criteria were verified. In-hospital controls were recruited from the departments of the ophthalmological clinic and the orthopaedic clinic of Bari and Taranto. The inclusion criteria for controls were those without a diagnosis of malignant neoplastic diseases, Acquired Immuno-Deficiency Syndrome (AIDS), eye diseases, thyroid disease, diabetic retinopathy, autoimmune diseases, allergic diseases, viral hepatitis, haematological preneoplastic diseases, such as monoclonal gammopathy of undetermined significance (MGUS), bone marrow aplasia, or myeloproliferative syndromes; similarly, patients undergoing organ transplants were not eligible for inclusion as controls. Cases and controls were recruited from within the same geographic area. The participation rates were 50% for the population controls, 80% for hospitalized controls, and 75% for cases. The population controls, who were selected from among healthy subjects from the regional health service database, were obviously less sensitized. Different methods of control recruitment in the case–control studies were studied, and the method based on the regional health system showed lower compliance (42%) than the method based on the involvement of general practitioners (57%) [18]. All participating cases and controls signed an informed consent form, which described the aims and methods of the study. They all provided a 40 ml blood sample for the biological portion of the study, which was used to study the gene/environment interaction with the assessment of biological exposure parameters (serum polychlorinated biphenyls [PCB], aryl hydrocarbons receptors [AHR], lymphocytic oxidative damage by COMET assay, different genetic polymorphisms, and other biological parameters). This part of the study will produce results only in the coming years.

### Questionnaire and exposure assessment by CAREX

All participating subjects were interviewed by a trained interviewer using a semi-structured questionnaire validated in the research project “Epilymph” and provided a complete work history. The questionnaire gathered information on socio-demographics; education; family history of cancer and specifically cancer of the haemolymphatic system; medical history; residential history; tobacco, alcohol and drugs use; work history; diet; physical activity; and reproductive history. We used general questionnaires and specific questionnaires for specific job activity.

The codes used to classify the job titles were allocated on the basis of qualitative crude definitions independent of matrices related to CAREX and contained in the questionnaires completed during the study. In this preliminary analysis, we used the CAREX database to assess occupational exposure to known and suspected carcinogens and pesticides. The same database was used in previous studies in Italy [19], Canada [20], Nicaragua and Panama [21]. The CAREX database sets exposure for occupational sectors. CAREX shows the frequency of reports of exposure to each of the 62 chemical and physical risk factors recognized by the International Agency for Research on Cancer (IARC) (Classes 1 and 2A) [19] for every sector. Every work activity obtained from our semi-structured questionnaires was attributed to an occupational sector that was coded by CAREX. A score was assigned to each risk factor from the report frequencies contained in the CAREX tables for that sector [0 = no exposure (no reports); 1 = low exposure (<75% of report frequency); 2 = medium-high exposure (>75% of frequency of reports)]. For each job activity, the duration (number of years) was recorded. This number was multiplied by the single score. For each subject were evaluated by one to five job activities, and the related products were added. The result of the sum of the products is the CEI. The procedure was based on the following formula:

$$ \mathrm{CEI}\ \left(\mathrm{carex}\right)=\varSigma\ \left(\mathrm{Ordinal}\  \mathrm{Score}\ast \mathrm{Years}\right) $$

Categorization of the CEI was as follows: CEI = 0 [no exposure (cumulative indicator = 0)]; CEI = 1 [low exposure (cumulative indicator < = 30)]; CEI = 2 [medium-high exposure (cumulative indicator >30)].

The CEI was not standardized because the statistics used were all non-parametric and therefore normalization was not necessary.

The analysis by job title was conducted only using the most recent job title, while the cumulative exposure was calculated over the entire work history.

Only the pathways of significant chemical substances were considered.

The power of the study was low, and the estimates, with an α type 1 error of 0.05%, were not steady but were equally significant.

### Statistical analysis

The statistical analysis was performed using the STATA 12 software, and it was mainly based on the use of non-parametric statistical distributions because of the non-Gaussian distributions of a large number of studied variables.

For the comparison of proportions, the distribution of Z was used as indicated in the “two-sample test of proportions calculator” procedure included in the abovementioned software.

The univariate analysis was based on the “tab odds” calculations for all the studied variables.

The multivariate analysis was instead based on the use of the “unconditional logistic model”, as indicated in the tables, the variables describing sister cancer familiarity, age at diagnosis, province, sex, pack/years (recoded) and level of education. No adjustment was made to the dietary habits because the univariate estimates showed no significant association with lymphoma.

## Results

Cases and controls were well distributed in the main categories of age, gender, residence, education level, and job title. However, they were predominantly more than 60 years old (43.7% of cases, 40.8% of controls), male (59.5% of cases, 60.5% of controls), residents of Bari (65.2% of cases, 67% of controls), high school graduates (34.2% of cases, 42.2% of controls), and blue collar workers (31.7% of cases, 31.6% of controls). Specifically, recruited individuals were mostly blue collar workers, clerks and agricultural workers. No significant difference was observed between cases and controls regarding these variables. The two groups were therefore perfectly comparable. The presence of doctors, nurses and researchers, although very low, was only observed among the cases. This finding was also described in the study of t’Mannetje et al. [22] (Table 1).

**Table 1:** Distribution of the main variables between cases and controls

		Cases		Controls		Proportions test	
Variables	Tot	n	%	n	%	z	P
Age							
Less than 20 years	7	6	3.8	1	1.3	0.5	0.3
21–40 years	55	37	23.4	18	23.7	0.0	0.5
41–60 years	72	46	29.1	26	34.2	−0.4	0.7
More than 60 years	100	69	43.7	31	40.8	0.3	0.4
Gender							
Females	94	64	40.5	30	39.5	0.1	0.5
Males	140	94	59.5	46	60.5	0.1	0.5
Province of residence							
Bari	154	103	65.2	51	67.1	−0.3	0.6
Taranto	67	44	27.9	23	30.3	0.2	0.4
Others	13	11	7.0	2	2.6	0.2	0.4
Title of study							
Degree	38	27	17.1	11	14.5	0.2	0.4
High school	86	54	34.2	32	42.1	0.6	0.3
Middle school	64	43	27.2	21	27.6	0.0	0.5
Primary school	46	34	21.5	12	15.8	0.4	0.3
Jobs							
Housewife	16	11	7.0	5	6.6	0.0	0.5
Physician	3	3	1.9	0	0.0	–	–
Blue collar	74	50	31.7	24	31.6	0.0	0.5
Nurse	1	1	0.6	0	0.0	–	–
Teacher	17	9	5.7	8	10.5	−0.4	0.6
Researcher	2	2	1.3	0	0.0	–	–
Craftsman/Merchant	13	11	7.0	2	2.6	0.2	0.4
Agricultural workers	18	14	8.9	4	5.3	0.2	0.4
White collar	43	28	17.7	15	19.7	−0.2	0.6
Military	10	5	3.2	5	6.6	−0.2	0.6
Student/Unemployed/Retired	3	3	1.9	0	0.0	–	–
Freelancer	8	2	1.3	6	7.9	−0.3	0.6
Technical	5	2	1.3	3	4.0	−0.2	0.6
Missing	21	17	10.8	4	5.3	–	–
Totals	234	158	100	76	100	–	–

Legend

Z = The z-score test for the two proportions is used when you want to know whether two groups differ significantly in some characteristics

### Univariate analysis

The analysis of lymphoma crude risk (ORs) for all 14 occupational activities was performed (Table 2), and only the highest estimates (agricultural activity) were used for the multivariate analysis. The same approach was also used to analyse all other studied factors.

**Table 2:** Distribution of crude risk (ORs) by occupational titles

	DLBCL			FL			CLL			SBCL			MM			NHL			HL			ALL LYMPHOMAS		
	OR	95% LCI	95% UCI	OR	95% LCI	95% UCI	OR	95% LCI	95% UCI	OR	95% LCI	95% UCI	OR	95% LCI	95% UCI	OR	95% LCI	95% UCI	OR	95% LCI	95% UCI	OR	95% LCI	95% UCI
Housewife	0.91	0.15	5.44	0.51	0.04	5.85	0.91	0.11	7.34	0.63	0.03	11.29	4.33	0.29	63.11	1.13	0.29	4.34	–	–	–	1.29	0.36	4.58
Physicians	–	–	–	–	–	–	–	–	–	–	–	–	–	–	–	–	–	–	–	–	–	–	–	–
Blue collars	0.67	0.22	2.07	1.31	0.42	4.12	1.31	0.45	3.75	0.47	0.06	3.29	0.6	0.08	4.1	1.01	0.47	2.13	2.47	0.59	10.39	1.15	0.56	2.36
Nurse	–	–	–	–	–	–	–	–	–	–	–	–	–	–	–	–	–	–	–	–	–	–	–	–
Teacher	0.42	0.07	2.9	1.02	0.15	6.62	1.33	0.28	6.16	–	–	–	1.01	0.06	16.2	0.89	0.28	2.79	–	–	–	0.51	0.17	1.55
Researcher	–	–	–	–	–	–	–	–	–	–	–	–	–	–	–	–	–	–	–	–	–	–	–	–
Craftsman/Merchant	5.74	0.26	124.6	8.39	0.77	9.13	1.99	0.11	34.09	17.87	0.32	380.63				3.91	0.44	34.74	–	–	–	3.6	0.4	32.05
Agricultural workers	9.35	1.99	43.6	–	–	–	2.01	0.36	11.03	–	–	–	8.16	0.7	54.15	2.44	0.64	9.28	–	–	–	2.05	0.54	7.75
Clerk	1.39	0.45	4.27	0.87	0.22	3.38	0.35	0.08	1.5	1.58	0.18	13.93	0.52	0.04	5.59	0.89	0.39	1.94	1.98	0.56	6.96	1.03	0.47	2.23
Military	–	–	–	1.11	0.17	6.9	0.53	0.07	3.69	4.16	0.25	68.81	–	–	–	0.51	0.13	1.94	–	–	–	0.45	0.12	1.72
Student/Unemployed/Retired	–	–	–	–	–	–	–	–	–	–	–	–	–	–	–	–	–	–	–	–	–	–	–	–
Freelancer	–	–	–	–	–	–	–	–	–	–	–	–	–	–	–	–	–	–	–	–	–	–	–	–
Technicians	–	–	–	–	–	–	1.62	0.19	13.84	–	–	–	–	–	–	0.74	0.09	5.85	–	–	–	0.49	0.06	3.85
Food operators	–	–	–	0.73	0.12	4.41	0.92	0.22	3.79	1.22	0.1	14.7	–	–	–	0.6	0.18	1.97	0.23	0.01	3.26	0.58	0.18	1.82

Legend: *HL* Hodgkin Lymphoma, *NHL* Non Hodgkin Lymphoma, *DLBCL* Diffuse Large B-Cell Lymphoma, *FL* Follicular Lymphoma, *CLL* Chronic Lymphocitic Leukemia, *SBCL* Single B Cell Lymphoma, *MM* Multiple Mieloma

Lymphoma risks were analysed for 22 chemical products; none of these was statistically significant. Only higher OR levels were observed for low/medium levels of butadiene [OR = 1.91 (0.68–5.38)]; low/medium levels of acrylonitrile [OR = 1.70 (0.60–4.83)]; low levels of ethylene dibromide [OR = 1.96 (0.59–6.44)]; low levels of ethylene dioxide [OR = 2.58 (0.52–12-64)]; low levels of formaldehyde [OR = 2.31 (0.76–7.02)]; low levels of nitrox dimethylamine [OR = 2.58 (0.52–12.64)]; low levels of toluidine [OR = 1.80 (0.63–5.08)]; medium/high styrene levels [OR = 1.65 (0.67–4.06)]; low levels of tetrachloroethylene [OR = 1.87 (0.83–4.24)]; low levels of trichloroethylene [OR = 1.45 (0.66–3.19)]; low levels of vinyl chloride [OR = 1.71 (0.69–4.21)]; low levels of PAH [OR = 20.31 (2.26–182.23)]; and medium/high PAH levels [OR = 12.50 (1.16–136.4)]. Crude risk associated with low [OR = 20.3 (2.3–182.2)] and medium-high [OR = 12.5 (1.2–134.4)] cumulative exposure to polycyclic aromatic hydrocarbons (PAHs) showed elevated risks, but they were based on very small numbers, and the very unsteady estimates were not used.

Crude risks were also analysed for 10 physical risk factors and none of these was statistically significant. Slightly higher levels of OR were observed only for: low glass wool levels [OR = 2.28 (0.59–8.73)]; low levels of ionizing radiation [OR = 1.78 (0.45–7.03)]; radon medium/high levels [3.28 (0.92–11.71)]; and high levels of solar radiation [OR = 1.76 (0.68–4.59)]. Crude risks were also analysed for seven heavy metals and no significant results were obtained for any of these. There was a higher crude risk only for cobalt [OR = 2.20 (0.62–8.01)]. The crude risks were analysed for nine drugs and no statistical significance was observed. Crude risks for 2 pesticides showed high OR values that were very close to statistical significance: high levels of Captafol [OR = 7.05 (0.90–56.16)]; low levels of dimethilsulfate (Paraquat) [OR = 2.48 (0.96–6.36)]. We also analysed the crude risks for 4 tumour familiarity that did not show any statistical significance; a higher OR was observed only for breast cancer familiarity [OR = 2.15 (0.77–5.99)]. As reported in several experiences [23–26], smoking (pack/years) was not a significant risk factor for lymphomas.

### Multivariate analysis

Table 3 shows the multivariate analysis that considered not only subjects with agricultural exposure but also industrial exposure to pesticides. Subjects exposed to Captafol showed a significant increase in risk for all lymphomas [OR = 2.4 (1.1–5.6)], in particular for NHL [OR = 2.6 (1.0–8.2)]. Subjects exposed to low levels of Paraquat also showed an increased risk for all lymphomas [OR = 2.9 (1.0–8.2)], particularly NHL [OR = 2.8 (1.0–8.2)]. Medium high exposure to radon was associated with risk for all lymphomas [OR = 9.5 (1.2–76.8)] and with NHL [OR = 8.8 (1.2–71.4)].

**Table 3:** ^a^ORs distribution of main types of lymphomas by different levels of cumulative exposure to selected study factors

Cumulative exposure	Lymphoma types														
	*All lymphomas*					*Hodgkin lymphomas*					*Non hodgkin lymphomas*				
	Cases	Controls	OR^a^	95% LCI	95% UCI	Cases	Controls	OR^a^	95% LCI	95% UCI	Cases	Controls	OR^a^	95% LCI	95% UCI
^b^ Captafol															
No	138	70	1	–	–	30	70	1	–	–	108	70	1	–	–
Low	6	5	0.73	0.2	2.69	0	5	–	–	–	6	5	1.03	0.27	3.89
Medium-high	14	1	–	–	–	0	1	–	–	–	14	1	–	–	–
Overall	158	76	*2.4*	*1.11*	*5.63*	*30*	*76*	1	–	–	128	76	*2.59*	*1.04*	*6.42*
Paraquat															
No	123	66	1	–	–	24	66	1	–	–	99	66	1	–	–
Low	28	6	*2.91*	*1.03*	*8.2*	*6*	*6*	1.95	0.38	10.04	22	6	*2.83*	*0.96*	*8.37*
Medium-high	7	4	1.1	0.26	4.59	0	4	–	–	–	7	4	1.27	0.3	5.41
Overall	158	76	1.51	0.8	2.87	30	76	1.52	0.35	6.58	128	76	1.52	0.79	2.94
Radon															
No	113	59	1	–	–	28	59	1	–	–	85	59	1	–	–
Low	26	14	0.97	0.44	2.1	2	14	0.12	0.01	1.25	24	14	1.2	0.54	2.65
Medium-high	19	3	*9.5*	*1.18*	*76.82*	*0*	*3*	0.12	0.01	1.25	19	3	*9.37*	*1.16*	*75.85*
Overall	158	76	1.71	0.97	3.02	30	76	0.12	0.01	1.24	128	76	*1.87*	*1.04*	*3.35*

^a^ All the estimates were adjusted by sister cancer familiarity, age at diagnosis, province, sex, packyears and level of education

^b^ For this cumulative exposure was difficult perform multiple analysis by exposure dummy variables

All the italicized values represent statistical significant estimates

Table 4 presents a significant association between overall exposure to Captafol with NHL [OR = 2.6 (1.1–8.2)] and with DLBCL subtype [OR = 5.3 (1.6–17.3)]. Low exposure to Paraquat was also associated with DLBCL [OR = 3.8 (1–15.3)] and FL [OR = 4.6 (1.1–20.2)] subtypes. Medium-high levels of exposure to radon were associated with DLBCL [OR = 13.7 (1.3–143.0)] and SBCL [OR = 64.4 (2.1–1959.6)].

**Table 4:** ^a^ORs distribution of Non Hodgkin lymphoma subtypes by different levels of Cumulative Exposure to selected study factors and agricultural occupation

CUMULATIVE EXPOSURE	NON HODGKIN LYMPHOMA					NON HODGKIN LYMPHOMA SUBTYPES																								
	*NHL*					*DLBCL*					*FL*					*CLL*					*SBCL*					*MM*				
	CASES	CONTROLS	OR^a^	95% LCI	95% UCI	CASES	CONTROLS	OR^a^	95% LCI	95% UCI	CASES	CONTROLS	OR^a^	95% LCI	95% UCI	CASES	CONTROLS	OR^a^	95% LCI	95% UCI	CASES	CONTROLS	OR^a^	95% LCI	95% UCI	CASES	CONTROLS	OR^a^	95% LCI	95% UCI
^b^ Captafol																														
No	102	70	1.0	–	–	25	70	1.0	–	–	22	70	1.0	–	–	38	70	1.0	–	–	8	70	1.0	–	–	9	70	1.0	–	–
Low	6	5	1.0	0.3	3.7	5	5	3.5	0.8	15.2	0	5	1.0	–	–	0	5	1.0	–	–	0	5	1.0	–	–	1	5	3.0	0.1	59.1
Medium-high	14	1	–	–	–	5	1	1.0	–	–	4	1	1.0	–	–	4	1	1.0	–	–	0	1	1.0	–	–	1	1	1.0	–	–
Overall	20	6	*2.6*	*1.1*	*8.2*	10	6	*5.3*	*1.6*	*17.3*	4	6	3.0	0.6	14.1	4	6	1.4	0.3	7.0	0	6	1.0	–	–	2	6	10.9	1.0	125.8
Paraquat																														
No	99	66	1.0	–	–	26	66	1.0	–	–	19	66	1.0	–	–	33	66	1.0	–	–	7	66	1.0	–	–	9	66	1.0	–	–
Low	22	6	2.8	0.9	8.2	7	6	*3.8*	*1.0*	*15.3*	6	6	*4.6*	*1.1*	*20.2*	7	6	3.5	0.8	16.1	1	6	–	–	–	0	6	1.0	–	–
Medium-high	7	4	1.1	0.3	4.8	2	4	1.6	0.2	12.3	1	4	1.1	0.1	13.8	2	4	0.9	0.1	6.8	0	4	–	–	–	2	4	3.3	0.2	63.8
Overall	29	10	2.1	0.9	5.3	9	10	2.7	0.7	9.6	7	10	3.3	0.9	12.3	9	10	2.9	0.8	10.3	1	10	1.3	0.1	14.7	2	10	2.0	0.1	26.9
Radon																														
No	85	59	1.0	–	–	22	59	1.0	–	–	17	59	1.0	–	–	29	59	1.0	–	–	4	59	1.0	–	–	8	59	1.0	–	–
Low	24	14	1.2	0.5	2.6	8	14	1.5	0.5	4.4	5	14	1.2	0.3	4.1	6	14	0.9	0.3	2.9	2	14	2.9	0.3	29.7	2	14	0.6	0.1	5.5
Medium-high	19	3	*8.8*	*1.1*	*71.4*	5	3	*13.7*	*1.3*	*143.0*	4	3	*12.7*	*1.2*	*137.2*	7	3	10.8	0.9	130.1	2	3	*64.4*	*2.1*	*1959.6*	1	3	*106.1*	*1.3*	*8620.0*
Overall	43	17	1.7	0.8	3.6	13	17	2.2	0.8	5.8	9	17	2.0	0.7	5.8	13	14	1.5	0.5	4.0	4	17	6.6	0.9	45.6	3	17	1.4	0.2	9.0
Agricultural occupation																														
No	98	68	1.0	–	–	23	68	1.0	–	–	25	68	1.0	–	–	34	68	1.0	–	–	8	68	1.0	–	–	8	68	1.00	–	–
Yes	14	4	2.4	0.6	9.3	8	4	*9.3*	*2.0*	*43.6*	0	4	1.2	0.7	2.0	4	4	2.1	0.4	11.0	0	4	1.0	–	–	2	4	6.16	0.70	54.15

^a^All the estimates were adjusted by sister cancer familiarity, age at diagnosis, province, sex, packyears and level of education

^b^For this cumulative exposure wasn’t possible perform multiple analysis by exposure dummy variables

Legend: *HL* Hodgkin Lymphoma, *NHL* Non Hodgkin Lymphoma, *DLBCL* Diffuse Large B-Cell Lymphoma, *FL* Follicular Lymphoma, *CLL* Chronic Lymphocitic Leukemia, *SBCL* Single B Cell Lymphoma, *MM* Multiple Mieloma

All the italicized values represent statistical significant estimates

Table 5 illustrates the association between agricultural occupations and the risk of different lymphoma subtypes. This occupation category was associated with DLBCL [OR = 10.9 (2.3–51.6)] and MM [OR = 16.5 (1.4–195.7)]. This finding is consistent with the study of Mester et al. [27].

**Table 5:** Association estimates (ORs^a^) between occupation as agricultural worker and different lymphoma subtypes

Agricultural worker							
		Total	No	Yes	OR	95% LCI	95% UCI
ALL LYMPHOMAS	No	72	68	4	1	–	–
	Yes	141	127	14	2.3	0.6	8.5
HL	No	72	68	4	1	–	–
	Yes	24	24	0	_	–	–
NHL	No	72	68	4	1	–	–
	Yes	117	103	14	2.7	0.7	10.1
NHL-DLBCL	No	72	68	4	1	–	–
	Yes	31	23	8	*10.9*	*2.3*	*51.6*
NHL-FL	No	72	68	4	1	–	–
	Yes	25	25	0	–	–	–
NHL-CLL	No	72	68	4	1	–	–
	Yes	37	33	4	2.4	0.5	13.3
NHL-SBCL	No	72	68	4	1	–	–
	Yes	8	8	0	–	–	–
NHL-MM	No	72	68	4	1	–	–
	Yes	10	8	2	*16.5*	*1.4*	*195.7*

^a^All the estimates were adjusted by sister cancer familiarity, age at diagnosis, province, sex, packyears and level of education

Legend: *HL* Hodgkin Lymphoma, *NHL* Non Hodgkin Lymphoma, *DLBCL* Diffuse Large B-Cell Lymphoma, *FL* Follicular Lymphoma, *CLL* Chronic Lymphocitic Leukemia, *SBCL* Single B Cell Lymphoma, *MM* Multiple Mieloma

All the italicized values represent statistical significant estimates

## Discussion

The observed association in this study between DLBCL subtype and agricultural occupations [OR = 10.9 (2.3–51.6)] is consistent with the results of a large pooled analysis of international studies [20, 26]. Moreover, general farm workers were at high risk of developing MM [OR = 16.5 (1.4–195.7)], as reported by Morton et al. [28].

A death certificate case–control study suggests that young agricultural worker residents from Southern Brazil were more likely to die from NHL than non-agricultural workers [29]. A meta-analysis suggested that total organo-chlorine pesticides (OCPs) was significantly positively associated with NHL risk [30].

Our study also showed a significant association between the occurrence of lymphoma and exposure to Captafol, which is used as a fungicide in agriculture, according to Mc Duffie et al. 2001 [OR = 2.51 (1.32–4.76)] [31]. In our data, a positive association was also observed with exposure to Paraquat, an herbicide, but there was an inverse trend with exposure level. Uncertainty in the interpretation of our findings might be related to the small study size and the crude definition of exposure. No data are available in the literature regarding the dose–response correlation of pesticides and lymphoma. Recently, however, some studies have reported a relationship between exposure to fungicides, herbicides or insecticides and NHL occurrence [32–35].

Moreover, sales of Captafol and Folpet, both fungicides, which have similar molecular structures, in the Apulia region increased from 80 kg to 741 kg [36] from 2002 to 2012, while sales of Paraquat in 2002 were 662 kg [37]. The extensive use of this pesticide in the Apulia region also explains its popular use for suicidal purposes [38] and horse poisonings [39]; in France, there was no apparent change in the number of suicide attempts involving Paraquat after its ban in July 2007 [40]. Captafol is a human carcinogen, in fact it was classified by the IARC as probably carcinogenic to humans (Group 2A). The Captafol production for use as a fungicide in the United States stopped in 1987. Its continued use from existing stocks was allowed, but in 1999 the Environmental Protection Agency banned its use on all crops except onions, potatoes, and tomatoes. In 2006 even these exceptions were disallowed, so currently its use on all crops is banned in the United States. Several other countries have followed suit since 2000, and as of 2010, no countries are known to allow the use of Captafol on food crops.

The carcinogenic mechanism of Captafol is attributed to its interaction with the thiol groups of glutathione and cysteine, which reduces the defence against oxidative agents, and the N-S bond formation with other biological substrates, both leading to the formation of metabolites such as tetrahydrophthalamide, which is considered a human carcinogen [41].

Paraquat (N,N′-dimethyl-4,4′-bipyridinium dichloride) is an herbicide widely used in agriculture. It is derived from the alkylating agent dimethyl sulphate.

Results from the Agricultural Health Study showed an increased risk of NHL among Paraquat-exposed pesticide applicators [42]. One study suggested that Paraquat increases superoxide dismutase activity and radiation resistance in mouse lymphoma cells [43]. Another study suggested that increased levels of metallothionein, glutathione S-transferase, Cu, Zn-SOD and Mn-SOD might be protective against Paraquat toxicity in acute myelogenous leukaemia (AML) cells [44]. Moreover, the possibility of Paraquat-induced DNA damage has been suggested [45].

Acute exposure to Paraquat accounted for several cases of fatal poisoning, while chronic exposure appears to be associated with respiratory disease and Parkinson’s disease [46]. Animal studies have shown DNA alterations in treated animals. In the past, exposure to Paraquat has been associated with melanoma, leukaemia, and cancer of the penis, cervix and lung. More recent studies found a significantly increased risk of developing NHL among subjects exposed to this substance. Therefore, our understanding of Paraquat carcinogenicity is limited, and further studies are warranted [41].

As indicated in Appendix 1 of the work of Mirabelli et al. [19], exposure to Radon, which was higher among agricultural workers, food and beverage production workers, and electricity workers, was also associated with DLBCL [OR = 2.5 (1.2–5.4)] and SBCL [OR = 5.7 (1.3–25.6)]. Radon is a product of the radioactive decay of radium. Radon is easily inhaled. The level of the Radon-gas hazard differs from location to location. Despite its short lifetime, radon gas from natural sources can accumulate in buildings, especially, due to its high density, in low areas such as basements and crawl spaces. Radon can also occur in ground water. Epidemiological studies have shown a clear link between breathing high concentrations of Radon and incidence of lung cancer. Radon is a contaminant that affects indoor air quality worldwide. According to the United States Environmental Protection Agency, Radon is the second most frequent cause of lung cancer, after cigarette smoking, causing 21,000 lung cancer deaths per year in the United States. As Radon itself decays, it produces other radioactive elements called Radon daughters (also known as Radon progeny) or decay products. Unlike the gaseous Radon itself, Radon daughters are solids and stick to surfaces, such as dust particles in the air. The Radon assessment was currently carried out in the studied Apulia areas and only in particular situations the concentration of Radon was above 300 Bq/m^3^. But for agricultural workers this exposure was prolonged in time. Moreover, there was suggestive, though statistically non-significant, evidence of a significant increase of DLBCL among children with a high residential indoor exposure to Radon [47] and an increased risk of CLL and HL incidence, and NHL mortality with increasing γ-ray dose among Uranium miners [48].

## Conclusions

This is a preliminary report of occupational risk factors for lymphoma in the provinces of Taranto and Bari (Apulia region, Southern Italy). Although limited in size and utilizing a crude method of retrospective exposure assessment, this work revealed that agricultural workers exposed to Captafol, Paraquat and radon could develop lymphoma subtypes, especially DLBCL. These findings confirm existing knowledge and suggest new hypotheses for research about occupational factors suspected to be associated with lymphoma risk.

One of the weaknesses of the study is the instability of the estimates even when they were significant. Such instability prompts us to be cautious about the conclusions even though they are consistent with previous studies.

## Abbreviations

AHR: Aryl hydrocarbons receptors
AIDS: Acquired immuno-deficiency syndrome
AIRC: Associazione Italiana per la Ricerca sul Cancro
AIRTUM: Associazione Italiana Registri Tumori
AML: Acute myeloid leukaemia
CAREX: CARcinogen EXposure
CEI: Cumulative exposure index
CLL: Chronic lymphocytic leukaemia
DIM: Interdisciplinary Department of Medicine
DLBCL: Diffuse large B-cell lymphoma
FL: Follicular lymphoma
GWAS: Genome-Wide Association Study
HL: Hodgkin Lymphoma
IARC: International Agency for Research on Cancer
MALT: Mucosa-Associated Lymphoma Tissue
MGUS: Monoclonal Gammopathy of Undetermined Significance
MM: Multiple myeloma
NHL: Non-Hodgkin Lymphoma
OCPs: Organo-Chlorine Pesticides
OR: Odds ratio
PAHs: Polycyclic aromatic hydrocarbons
PCB: Polychlorinated biphenyls
PRIN: Research Project of National Interest
SBCL: Small B-cell lymphoma
WHO: World Health Organization
