# Correction to: Risk of lymphoma subtypes by occupational exposure in southern Italy

**DOI:** 10.1186/s12995-020-00256-1

**Published:** 2020-03-20

**Authors:** Giovanni Maria Ferri, Giorgina Specchia, Patrizio Mazza, Giuseppe Ingravallo, Graziana Intranuovo, Chiara Monica Guastadisegno, Maria Luisa Congedo, Gianfranco Lagioia, Maria Cristina Loparco, Annamaria Giordano, Tommasina Perrone, Francesco Gaudio, Caterina Spinosa, Carla Minoia, Lucia D’Onghia, Michela Strusi, Vincenzo Corrado, Domenica Cavone, Luigi Vimercati, Nunzia Schiavulli, Pierluigi Cocco

**Affiliations:** 1 Department of Interdisciplinary Medicine (DIM), Section “B. Ramazzini”, Regional University Hospital “Policlinico - Giovanni XXIII°”, Unit of Occupational, Medicine, University of Bari, Piazza G. Cesare, 11, 70124 Bari, Italy; 2 Department of Emergency and Transplantation (DETO), Regional Universitary Hospital “Policlinico - Giovanni XXIII°, Unit of Hematology, University of Bari, Piazza G. Cesare, 11, 70124 Bari, Italy; 3 ASL Taranto, Moscati Hospital, Unity of Haematology, Via Paisiello 1, 74100 Taranto, Italy; 4 Department of Emergency and Transplantation (DETO), Regional University Hospital “Policlinico – Giovanni XXIII° ”, Unit of Pathology, University of Bari, Piazza G. Cesare, 11, 70124 Bari, Italy; 5 Department of Public Health, Clinical & Molecular Medicine, Occupational, Health Section, University of Cagliari, 09100 Cagliari, Italy; 6 Interdisciplinary Department of Medicine (DIM), University Hospital. Policlinico-Giovanni XXIII, University of Bari, Piazza Giulio Cesare, 11, 70124 Bari, Italy

**Correction to: J Occup Med Toxicol**

**http://dx.doi.org/10.1186/s12995-017-0177-2**

After publication of our article [1] we have been notified that one of the author names has been incorrectly spelled.

- Original name spelling:
  Guadio F.
- Correct name spelling:
  Gaudio F.
